# Smoking cessation program preferences of individuals with chronic obstructive pulmonary disease: a qualitative study

**DOI:** 10.1017/S1463423624000306

**Published:** 2024-09-20

**Authors:** Noah Tregobov, Kassandra Starnes, Saron Kassay, Maryam Mahjoob, Yu seon Sarah Chae, Austin McMillan, Iraj Poureslami

**Affiliations:** 1 Vancouver-Fraser Medical Program, University of British Columbia, Vancouver, Canada; 2 Faculty of Medicine, Respiratory Medicine Division, University of British Columbia, Vancouver, Canada; 3 Faculty of Law, University of Victoria, Victoria, Canada; 4 Faculty of Health Sciences, Simon Fraser University, Burnaby, Canada; 5 Schulich School of Medicine & Dentistry, Western University, London, Canada; 6 Canadian Multicultural Health Promotion Society, Vancouver, Canada

**Keywords:** chronic obstructive pulmonary disease, e-cigarette, telehealth, tobacco cessation, smoking cessation

## Abstract

**Aim:**

To explore the views of tobacco-smoking chronic obstructive pulmonary disease (COPD) and asthma-COPD overlap (ACO) patients on telehealth-based cessation programs and the role of e-cigarettes as an aid to quit smoking.

**Background:**

Tobacco smoking accelerates the progression of COPD. Traditional smoking cessation programs often do not entirely address the unique needs of COPD patients, leading to suboptimal effectiveness for this population. This research is aimed at describing the attitudes and preferences of COPD and ACO patients toward innovative, telehealth-based smoking cessation strategies and the potential application of e-cigarettes as a quitting aid.

**Methods:**

A qualitative exploratory approach was adopted in this study, employing both focus groups and individual interviews with English-speaking adults with diagnosed COPD or ACO. Participants included both current smokers (≥ 5 cigarettes/day) and recent ex-smokers (who quit < 12 months ago). Data were systematically coded with iterative reliability checks and subjected to thematic analysis to extract key themes.

**Findings:**

A total of 24 individuals participated in this study. The emergent themes were the perceived structure and elements of a successful smoking cessation program, the possible integration of telehealth with digital technologies, and the strategic use of e-cigarettes for smoking reduction or cessation. The participants stressed the importance of both social and professional support in facilitating smoking cessation, expressing a high value for insights provided by ex-smokers serving as mentors. A preference was observed for group settings; however, the need for individualized plans was also highlighted, considering the diverse motivations individuals had to quit smoking. The participants perceived online program delivery as potentially beneficial as it could provide immediate access to support during cravings or withdrawals and was accessible to remote users. Opinions on e-cigarettes were mixed; some participants saw them as a less harmful alternative to conventional smoking, while others were skeptical of their efficacy and safety and called for further research.

## Introduction

Tobacco use results in over 7 million global deaths yearly and is a predominant risk factor for chronic obstructive pulmonary disease (COPD) (World Health Organization, n.d.; Gakidou *et al*., 2017). Smoking cessation is paramount for slowing COPD progression (Polosa *et al*., 2018; Centers for Disease Control and Prevention, 2019; Polosa *et al*., 2020) and offers improved quality of life (Jimenez-Ruiz *et al*., 2018), reduced mortality (Bai *et al*., 2017; Global Initiative for Chronic Obstructive Lung Disease, 2020), enhanced lung function (Pezzuto *et al*., 2018; Pezzuto and Carico, 2019; Global Initiative for Chronic Obstructive Lung Disease, 2020), and symptom relief (Pezzuto *et al*., 2018; Global Initiative for Chronic Obstructive Lung Disease, 2020). However, long-term abstinence remains a challenge due to habit-breaking difficulties, insufficient patient-provider rapport (van Eerd *et al*., 2017), lack of practical education, and withdrawal symptoms (Jiménez-Ruiz *et al*., 2001; van der Meer *et al.,* 2003; Eklund *et al.,* 2012; Livingstone-Banks *et al*., 2019). Traditional cessation programs often emphasize self-help/coping strategies enriched with pharmacotherapy and counseling (Centers for Disease Control and Prevention, 2019); however, they are not always tailored to the needs of individuals from certain populations and communities.

Emerging digital technologies and telehealth interventions facilitate virtual delivery of smoking cessation programs, offering increased accessibility and potential cost reduction (Taylor *et al*., 2017; Hallensleben *et al*., 2019; Haluza *et al*., 2020; Shoenbill *et al*., 2022). Dahne *et al*. (2022) reported that a virtual cessation approach increased pharmacotherapy adherence for individuals trying to quit smoking compared to telephone-based counseling. Still, the effectiveness of virtual interventions depends on individual access to and comfort with using technology, as well as provider proficiency (Haluza *et al*., 2020; Cobos-Campos *et al*., 2023). Therefore, understanding user perceptions is pivotal for optimizing such interventions (Taylor *et al*., 2017).

Electronic cigarettes (e-cigarettes) and vaping products are devices that deliver nicotine or other substances to users in a vaporized form and are viewed by some as smoking cessation tools (West *et al*., 2016; Filippidis *et al*., 2019). In 2020, the prevalence of e-cigarette usage stood at 11% globally but reached 43% among smokers (Tehrani *et al*., 2022). Although associated risks exist (Anderson *et al*., 2016; Bhatta and Glantz, 2019; Kligerman *et al*., 2020; Werner *et al*., 2020), some studies show e-cigarettes might be beneficial for COPD smokers seeking harm reduction (Morjaria *et al*., 2017). Determining the role of e-cigarettes in cessation strategies requires understanding patients’ attitudes toward these devices (Morjaria *et al*., 2017; Farsalinos, 2017).

While the benefits of smoking cessation are well-recognized, standard interventions often do not meet the specific needs of individuals with chronic conditions like COPD, who face unique challenges when quitting (Feng *et al*., 2022). Recognizing the urgent need for practical, customizable strategies (Ho *et al*., 2021), this study seeks to address this gap by exploring the perspectives of tobacco-smoking COPD and asthma-COPD overlap (ACO) patients on emerging topics in smoking cessation. Specifically, we investigated participants’ views on the use of telehealth in cessation efforts and their attitudes toward e-cigarettes as a potential alternative to traditional quitting methods. The research questions are: *What are the essential components of effective smoking cessation programs as perceived by individuals with COPD or ACO? And what alternative cessation methods do individuals with COPD or ACO consider beneficial?* The insights gained from this study aim to facilitate patient-centered cessation strategies, catering to the preferences and needs of this demographic.

## Methods

We used the COnsolidated criteria for REporting Qualitative research, a checklist for reporting qualitative research (Tong *et al*., 2007) (Appendix 1).

### Overview & study design

This study was conducted in 2019 in Vancouver, Canada, using a qualitative exploratory design (Stebbins, 2001). We employed a thematic analysis within the framework of Qualitative Grounded Theory to systematically explore and conceptualize the experiences and needs of COPD and ACO patients in smoking cessation, aiming to inform the development of tailored, patient-centered cessation strategies. Data were sourced from two focus groups and two individual interviews. The study received ethics approval from the University of British Columbia Office of Behavioural Research Ethics (H12-03689).

### Participant eligibility & recruitment

Inclusion criteria were English-speaking adults (≥ 19 years), either current smokers (≥ 5 cigarettes/day) or recent ex-smokers (< 12 months) with a diagnosis of COPD or ACO. Participants were recruited through convenience sampling from collaborating lung clinics and pulmonary rehabilitation programs in the Greater Vancouver Area, along with database of lung clinic patients who had agreed to be contacted for future research opportunities. A research assistant contacted potential participants via both email and telephone to share information about the study, verify eligibility, and determine their willingness to participate.

### Focus group & interview content development

For the development and validation of the focus group and interview content, we conducted a thorough literature review on smoking cessation approaches, incorporated both professional and patient perspectives, and drew insights from prior studies (FitzGerald *et al*., 2015; Poureslami *et al*., 2015; Poureslami *et al*., 2020; Tregobov *et al*., 2020). The initial questions were then refined in collaboration with cessation experts from the Vancouver Coastal Health (VCH) Tobacco Control Clinic and the BC Lung Association. While the interview guide covered four key topics, for the scope of this manuscript, we concentrated on: *Topic 2) The structure of a smoking cessation program and Topic 4) The feasibility and application of alternative cessation methods, including e-cigarettes and telehealth*. It’s important to note that insights from: *Topic 1) Risk perceptions, attitudes, and beliefs regarding smoking and Topic 3) The impact of smoking on health* were part of a separate project with distinct research questions, but are noted in this manuscript for completeness. The full interview guide can be found in the Appendix.

### Focus group and interview structure, procedures, and data collection

The semi-structured focus groups (Britten, 2006) were conducted by a male author, I.P., who holds a PhD and is a senior health evaluation research scientist with extensive experience in the field. With over 25 years of experience in various research methodologies, including qualitative studies, I.P. brought a significant depth of knowledge to the research process. Some participants may have been previously acquainted with I.P. through their involvement in prior smoking cessation and lung health studies. For those participants who were unable to attend the in-person focus group session at the research center, individual interviews were arranged. Participants provided informed, written consent before sessions. Content questions were discussed openly, with the facilitator also ensuring all participants had an opportunity to share their thoughts prior to moving on to the next topic. Group sessions and individual interviews were audio recorded, transcribed, and de-identified by members of the research team. Observational/field notes were also taken by study facilitators to facilitate contextualization of participants’ data and served as a reference point during analyses. Focus group durations were approximately 90 minutes, while individual interviews took about 30 minutes to complete. Participants received a $30 CAD stipend to reimburse their travel and parking expenses.

### Data analysis

Data were reviewed independently by two research team members. Guided by the research questions, a primary coding guide was established and discussed by the team. Initial coding was conducted using NVivo software (QSR International, Version 12). An inter-coder reliability check between a member of the research team and the senior health evaluation research scientist reached 94% agreement; ≥80% is considered an acceptable level of agreement (O’Connor and Joffe, 2020). Following the reliability check, the team member coded the remaining transcripts, with definitions iteratively revised to ensure contextual relevancy, including through referencing observation notes. Throughout coding, the corresponding author reviewed every fifth code, and a 90% agreement was achieved on remaining transcripts. Subsequently, the transcripts and all assigned codes were thoroughly reviewed by the team to check for accuracy. Any discrepancies were addressed by a mediator from the research team. The codes were then analyzed and placed into higher-level nodes and subsequently grouped into themes. This paper discusses three emergent themes related to the research questions, as explained in the Findings section.

## Results

In September and October of 2019, 78 individuals were contacted, and 47 individuals were eligible. From this group, 22 individuals attended one of two focus group sessions (10 and 12 participants per session) and the remaining 25 individuals could not attend the group sessions due to various constraints such as scheduling conflict or transportation limitations. Amongst those who did not attend a group session, two participants were interviewed individually (using the same interview guide).

All 24 participants had made three or more attempts to quit smoking in the past. Sixteen males and eight females participated, and their ages spanned from 40 to 80 years. Participant demographics are detailed in Table 1.

**Table 1.** Participant characteristics (n = 24)

**Self-reported gender**	
Female [n(%)]	8 (33.3)
Male [n(%)]	16 (66.7)
**Condition**	
ACO [n(%)]	10 (41.7)
COPD [n(%)]	14 (58.3)
**Smoking status**	
Current smoker [n(%)]	13 (54.2)
Ex-smoker [n(%)]	11 (45.8)

### Findings

Participants discussed numerous topics related to smoking and cessation based on their knowledge, perceptions, and lived experiences (refer to Appendix). Three main themes emerged: (1) the structure and elements of an effective smoking cessation program; (2) the integration of telehealth and digital technologies in cessation programs; and (3) the utilization of e-cigarettes for smoking reduction or cessation. Participants’ quotes for each specific theme are summarized below and additional quotes for each theme can be found in Table 2.

**Table 2.** Participants quotes

**Theme 1:** *The structure and elements of an effective smoking cessation program*	‘if they *[ex-smokers]* could come and tell their story and how they did it and you think ’well if they can do it why can’t I do it,’ even though everybody’s different’. ‘I did not even know that there was such a program…’ ‘I found it very helpful [*to have ex-smokers participate*], the people that had been there done that kind of people, that for me a lot of times, you’re getting a lecture you’re hearing about the perils, you’re hearing it from someone you don’t give a lot of credibility to’. ‘Well, I think it’s like seeing the success of other people, being able to hear their stories, how they quit, you know? And just being able to have hope, you know?’ I would love to be in a class that talks about it *[quitting smoking]*. I have some friends who talk about it all the time. They encourage me all the time, and if it wasn’t for them, I would not still be quit’. ‘I liked them both *[individual and group sessions]* equally because the individual attention we could talk about stuff that was bothering me…the group was good for my support’. ‘AA [Alcoholics Anonymous], the people that drinking is their issue, they’ve got that concept of you need to have a mentor within the group and for support, and if you’re going to lapse there’s oh here’s my lifeline that I can get support from’. ‘…not a pamphlet nothing on the walls, like if I wasn’t in this group I wouldn’t know about this [smoking cessation group]…my doctor doesn’t know about it…a lot of people want help but they don’t know that help is available’.
**Theme 2:** *The integration of telehealth and digital technologies in cessation programs*	‘I frequently go on a subreddit website called Reddit stopsmoking and it’s all just people who are bringing up discussions or trying to motivate others or answer people’s questions or get people through hard times, and all you need is an internet connection’. ‘…there’ll always be somebody there, cause that moment you reach for that cigarette, that’s when you got to make that decision…the moment you open that pack, are you going to take one or not? If it clicks, you can talk to somebody about it’. ‘’…the entire history of the world fits in the pocket of your pants or jacket so why not utilize that for people who only have ten minutes in between appointments and so they can make a quick phone call or Facebook group or Reddit group or anything and reach out for support’. ‘I’ve used the Medeo and the Telus health…I used it for a while for prescription renewals and basic stuff. I found it to be great, and easier when I was on the couch and felt like crap’. ‘wouldn’t it be better if there was something available all the time on an ongoing basis? An open resource’.
**Theme 3:** *The utilization of e-cigarettes for smoking reduction or cessation*	‘It’s stupid, you’re still smoking *[when using e-cigarettes]*. You can change the name, it’s still the same thing. Your either smoking or you’re not and that’s just it, and there are people dying, they’re banning it in the states *[United States]*, and they’re not doing it for nothing’. ‘I don’t want to say what works for me would work for everyone, but for myself and my medical conditions, I have a lot less breathing restrictions in the morning or while using an e-cigarette than I did with cigarettes. I still do have some *[breathing restriction]*, it’s not a catch-all, but it is, in my experience, mitigated’. ‘I think it’s cool in that it’s a healthier alternative if someone is trying to quit. I have a respect for it *[vaping]*. I like that it exists because I know in my father’s case, and people who are stuck, they can’t quit. So if they can still save their health somehow that’s like a really cool thing’. ‘The feeling of the nicotine somehow running through our blood and if we hope that we’re mitigating the health concerns we’re not thinking let’s put it that way frankly. It’s doing the job it keeps us happy and hopefully it’s less problematic than smoking hopefully’. ‘I tried vaping. to quit smoking. I found that I sucked the vapour in deep into my lungs, right, and I still wasn’t getting the nicotine adrenaline thing – so I ended up vaping and smoking’.

### Theme 1: The structure and elements of an effective smoking cessation program

Participants shared their smoking histories, patterns, and cessation experiences, while also discussing ways to enhance and improve current cessation resources and services.

A prevalent sentiment was the importance of both social (e.g., friends, peers) and professional (e.g., clinicians, educators) support, and its accessibility during the quitting process: *‘I, as an addict, I need that support. Yes, it’s fine to go through this [group session], but now I’m going to leave… I’m on my own’.*

The potential role of ex-smokers as mentors during cessation was emphasized, with many wanting insights from those with firsthand lived experience: *‘Hearing other people’s stories about how they’ve quit would be immensely helpful’.* The relatability of ex-smokers was highlighted*: ‘Ex-smokers are easier to relate to, they know what you’re going through’.* The emphasis was on application of valuable strategies used by ex-smokers in smoking cessation attempts: *‘I think that the [ex-smokers] that I was dealing with in [a prior smoking cessation] program have a lot of credibility and were helpful certainly in getting the wheels moving as to a couple of different techniques, tools to try’.*

Participants debated individual versus group-based cessation approaches. Group settings were generally favored for shared experiences and peer support: *‘Yeah, I think this kind of a [group cessation] forum with several different people orchestrating it is helpful because you’ve all got your different perspectives…so I think this kind of a setting would be more helpful than the one-on-one [format]’.* Another added: *‘I think that would be great…people could encourage one another’.* However, the significance of individualized cessation plans was also emphasized by some participants: *‘I think the community is great, however, we are also seeing that each one of us has a different reason to quit…[a cessation plan] needs to become individualized’.* A concern about group settings potentially triggering cravings for smoking was also shared: *‘There’s a flip side to [using a group approach to quit] – the group may help quit, but it could go the other way too…the talking makes you think about [smoking]’.*

### Theme 2: The integration of telehealth and digital technologies in cessation programs

Participants largely viewed telehealth and digitial technologies favorably for smoking cessation. A main perceived advantage was immediate and convenient access during cravings or moments of difficulty: *‘If [smokers] just have a quick question, they can get a response immediately…as opposed to [having to] wait for their next [consultation] which is two to three days from now. What do you think is going to happen in those two to three days? They’re probably going to open that pack’.* Another participant valued this digital interface due to the stigma associated with smoking: *‘If I could have face-timed or video chatted with a support worker, my earlier [quitting] attempts would have been far more successful. There’s so much shame attached to being a smoker these days…it’s hard to talk to your friends, especially if they’ve never smoked’.*

The notion of timely contact during cravings was emphasized as comforting: *‘I think having [telecommunication] and knowing you have somebody there to talk to you [is important]’.* Another added: *‘Access [to support] if you are in a position where you feel you’re going to fail or drop off, you need some support right now to be able to communicate to somebody’.* However, a few participants questioned whether telecommunication without the in-person interactions would be meaningful in smoking cessation: *‘I’m not sure an app would make a big difference, you need the physical’.*

Many viewed telehealth as especially valuable for underserved or remote smokers: *‘I would absolutely say that any form of communication, especially for people living in rural areas, who don’t have a lot of interpersonal connection, it can be great’.* Virtual support groups (e.g., via Zoom meeting) were also proposed as a cost-effective approach: *‘You could just join in, up to twelve people easy. It’s not expensive’.*

### Theme 3: The utilization of e-cigarettes for smoking reduction or cessation

Participants offered diverse views on e-cigarettes as alternatives to traditional smoking cessation methods. Many, particularly those with experience using e-cigarettes, perceived vaping as less harmful than traditional cigarettes: *‘I don’t consider it to be as serious as smoking a cigarette, for sure’.* Another said: *‘We still feel that [vaping is] better, it’s not the best…Quitting is the best, but it’s better at this point than smoking’.* In contrast, those generally more unfamiliar with e-cigarettes expressed uncertainty about potential adverse outcomes of vaping. One argued: *‘If I did the vaping…now instead of the cigarettes, I think it would have done a lot more damage to the lungs more quickly than the cigarettes’.*

The conversation frequently shifted to the composition and harm of e-cigarettes versus cigarette smoke. A participant noted: *‘[E-cigarette smoke] doesn’t have all the carcinogens. It has the nicotine in there, so you’re still hooked on something, but you’re not getting all the carcinogens’.* Another compared: *‘I believe that [vaping] is the lesser of two evils and I think there is something like seven-thousand different chemicals in a cigarette and something like seven hundred in a vape’.*

Opinions varied on e-cigarettes’ role in cessation. Some highlighted the possibility of reducing nicotine content over time: *‘I feel if you’re trying to quit, it actually is useful because you can get ones with progressively less nicotine’.* Another supported this: *‘And on the e-vaping thing again, I do know a number of people who have quit by going down [in nicotine dose], they have done it’.* Yet, another raised concerns about e-cigarettes’ nicotine content: *‘[E-cigarette smoke is] high nicotine though, and when you smoke it a lot you end up being more addicted to nicotine, more than you normally would’.*

A few participants advocated for further research. One suggested: *‘I think if you could prove that vaping is controlled… if you could prove that it was not detrimental to your lungs, I would think that would be a valid way to go to try to get people off of traditional cigarettes’.* Another cautioned against potential societal impacts of normalizing e-cigarettes: *‘No, I don’t think it’s going to be a way to [quit]… it’s going to make nicotine addiction more appealing to youth’.*

## Discussion

In this qualitative study, we explored the perceptions of respiratory patients (COPD and ACO) regarding the structure and features of an effective smoking cessation program. The majority of participants emphasized the importance of a support network that extends beyond professionals, highlighting the perceived value of peers, friends, and family in providing emotional support, accountability, and encouragement throughout the cessation process (Barnes *et al*., 2020). This study also contributes to the existing body of knowledge surrounding the perceived importance of non-professional social supports in the smoking cessation process. The influence of social networks on successful quitting is supported by van den Brand *et al*. (2019) and literature stressing the heightened intent to quit smoking when backed by social and community support (Carlson *et al*., 2002; Meijer *et al*., 2016; Patten *et al*., 2016; Soulakova *et al*., 2018). The potential of an interdisciplinary approach, encompassing family, professionals, and peers, holds promise for fulfilling diverse support needs (Poureslami *et al*., 2014; Campbell *et al*., 2008; Ford *et al*., 2013). Participants highly endorsed group-based sessions for their potential to nurture shared experiences, collective learning, and peer motivation. It may be advantageous for program administrators to integrate structured peer-led support groups into smoking cessation programs, leveraging the collective strength and shared experiences of individuals on their quit journey. This communal approach, supported by evidence from Stead *et al*.’s meta-analysis (2017), can strengthen self-perception, learning from lived experiences, and positivity (Jenks, 1994).

The involvement of an ex-smoker as a peer supporter in a smoking cessation journey emerged as a key finding. Ex-smokers’ firsthand lived experience and personal relatability, more so than professional staff, were seen as beneficial in addressing queries, sharing insights from their cessation journey, and offering pertinent advice on managing cravings and withdrawal symptoms. Such peer involvement not only aligns with the literature emphasizing empathetic peer support (Campbell *et al*., 2008; Westmaas *et al*., 2010; Ford *et al*., 2013), but also underscores their role in reducing smokers’ feelings of isolation, bolstering their ability to manage addiction, and enhancing their motivation and confidence in their smoking cessation endeavors (Westmaas *et al*., 2010; Williams *et al*., 2010; Zhao *et al*., 2016). Considering these insights, health professionals and policymakers should consider the role of ex-smokers in peer support capacities, acknowledging their potential to contribute meaningfully to the smoking cessation process.

Participants recognized the potential benefits of integrating telehealth into smoking cessation programs. A primary advantage of telehealth or virtual modalities is the immediate access to support, a finding consistent with Haluza *et al*. (2020). Knowing that real-time access is available during challenging moments or intense cravings could alleviate anxiety and stress for some individuals. Participants also highlighted the possibility of facilitating support groups or networks through virtual platforms or apps, with online peer exchanges and daily automated messages having shown effectiveness in engaging smokers and fostering online support communities (Pechmann *et al*., 2015). Such digital interventions reportedly enhance feelings of provider support and overall motivation for quitting (Liebmann *et al*., 2019). Moreover, telehealth may improve accessibility for underserved or remote areas where smoking prevalence is notably high (Roberts *et al*., 2016; Buettner-Schmidt *et al*., 2019; Bhaskar *et al*., 2020). However, despite reported advantages of telehealth in smoking cessation, concerns were expressed regarding the potential decline in personal interactions with an increased digital focus. In addition, for elderly participants, virtual programs could alleviate physical access challenges, although other challenges may still arise such as inadequate digital literacy and lack of access to relevant devices (Arcury *et al*., 2018; Bhaskar *et al*., 2020; McGee *et al*., 2020; Merianos *et al*., 2021; Kotsen *et al.,* 2021). While virtual cessation platforms present an avenue for broader service delivery, it is crucial to tailor them to the unique lifestyles, capabilities, and needs of target populations (Phillips and McLeroy, 2004). Overall, our findings suggest that a nuanced approach to telehealth, which considers unique challenges such as digital literacy and access issues, could bridge critical service gaps in current cessation efforts, especially in underserved or remote areas.

Discussions on the merit of using e-cigarettes as a smoking cessation tool elicited mixed opinions. Reflecting on these varied viewpoints, our study advocates for the inclusion of patient experiences and preferences in the development of cessation tools, thereby aligning strategies with patient needs and the complex realities of quitting smoking. Participants with e-cigarette experience largely viewed them as less harmful than traditional tobacco and appreciated the adjustable nicotine levels, which could facilitate gradual nicotine reduction. The perceived benefits of e-cigarettes reported in literature include suitability for indoor use (Hanafin and Clancy, 2020), enhanced social acceptability (Simmons *et al*., 2016), and anecdotal improvements in respiratory health, including in COPD patients, upon transitioning to e-cigarettes (Morjaria *et al*., 2017; Singh *et al*., 2017). Conversely, participants less familiar with e-cigarettes voiced concerns about potential increased tolerance and dependence. Some research suggests that smokers perceive nicotine-containing e-cigarettes as more addictive than conventional cigarettes (Jankowski *et al*., 2019; Hanafin and Clancy, 2020). While positive attitudes toward e-cigarettes correlate with successful cessation attempts in some studies (Harrell *et al*., 2015; Rutten *et al*., 2015), others have found that e-cigarette use, regardless of motivation, might reduce the chances of quitting smoking (Kalkhoran and Glantz, 2016; Patil *et al*., 2019). Consequently, individuals’ perceptions and prior experiences with cessation tools, including e-cigarettes, should be included in program development. The known and possible undiscovered harms of e-cigarettes must be considered and communicated against their potential benefits for some individuals. Further patient-centered research like stakeholder interviews, scoping reviews, and analysis of administrative datasets is needed. This research could improve our understanding of how e-cigarettes might be utilized as a population health tool to reduce smoking morbidity and mortality and identify research and practice gaps, which can guide the creation of tailored smoking cessation programs for diverse population groups.

This study has several limitations. Patients were primarily sourced from clinical settings, potentially skewing their smoking and cessation-related perspectives compared to the broader population we target. The sample size of 24 participants was determined by the availability and willingness of participants to engage in the study, characteristic of a convenience sample. While saturation was not assessed, the findings form a base for the conduction of further investigations, especially around the views and preferences of COPD and ACO patients to guide further research. Additionally, the utilization of both focus groups and individual interviews, while providing a means to include a wider array of participant experiences and accommodate individual availability, introduced a potential limitation by adding variability in response depth and context, which could influence the comparability and consistency of the data analysis. Next, the focus group format might have influenced social desirability and response biases (Barbour and Kitzinger, 1999; Grimm, 2010; Nyumba *et al*., 2018), especially when discussing sensitive topics like cessation challenges. Despite this, we endeavored to create an open dialog, with many participants later expressing appreciation for the discussions and comfort in connecting with peers with similar smoking and quit attempt experiences. Language barriers might have also influenced participation since sessions were conducted in English. Moreover, socioeconomic status of patients was not considered in this study despite potential associations between lower socioeconomic status and cigarette use (Hiscock *et al.,* 2012). This omission could restrict the generalizability of the results to populations with more varying socioeconomic backgrounds. We recognize that larger studies engaging a broader participant base would not only corroborate these findings but also enhance the generalizability to other disease contexts and the general population. For future work, engaging individuals from varied ethnocultural backgrounds is crucial to ensure culturally and linguistically appropriate care (FitzGerald *et al*., 2015; Poureslami *et al*., 2015; Poureslami *et al*., 2020; Tregobov *et al*., 2020), acknowledging that the dynamics of smoking cessation can differ across cultural and socioeconomic spectrums (Nguyen-Grozavu *et al*., 2020; Thomson *et al*., 2022).

This study provides insights for a patient-centered smoking cessation program focusing on practicality, accessibility, and relevance. Patients’ voices should be considered in framework development, including aspects such as: (1) engagement of ex-smokers as mentors, alongside family and peers for support; (2) group sessions for knowledge dissemination; and (3) digital access to essential resources and services. Such innovations aim to provide timely resources and align with the needs articulated by our study’s participants. Given the preliminary nature of this study, more evidence is required to validate the practical integration of these strategies.

In our future research endeavors, we aim to delve into two broad areas. Our focus will be a gap analysis to identify shortcomings in the field through a scoping review of programs and review of administrative/gray documents. Subsequently, our attention will shift to qualitative studies, seeking to understand the perceptions of specific demographic groups of smokers in relation to e-cigarettes and potential cessation techniques. This exploration will also encompass investigations into the potential of select models to bring about changes in smoking behaviors, utilizing a diverse range of methods.

## Conclusion

Our study describes the perspectives of COPD and ACO patients on smoking cessation programs. Participants have identified that cessation efforts might be improved through personalized support mechanisms, tailored resource access, and the integration of innovative approaches such as telehealth. Additionally, our findings shed light on the complex attitudes patients hold toward e-cigarettes, weighing potential benefits against concerns. Such nuanced viewpoints are crucial in designing patient-centered cessation strategies that not only meet but are also shaped by the specific needs and preferences of the COPD and ACO smoking populations.

## Supporting information

Tregobov et al. supplementary material 1 Tregobov et al. supplementary material

Tregobov et al. supplementary material 2 Tregobov et al. supplementary material

## Supplementary material

To view supplementary material for this article, please visit https://doi.org/10.1017/S1463423624000306
