# Supplementary material for: Smoking cessation program preferences of individuals with chronic obstructive pulmonary disease: a qualitative study
Source: Prim Health Care Res Dev. 2024 Sep 20;25:e38. doi: 10.1017/S1463423624000306 (PMC11464802; doi:10.1017/S1463423624000306)
Supplement: Tregobov et al. supplementary material 2 — Tregobov et al. supplementary material [file S1463423624000306sup002.docx]

**Focus Group Questions**

**Topic 1) Risk perceptions, attitudes, and beliefs regarding smoking**

- What does smoking mean to you?

- From your point of view, do you think smoking is a habit, addiction, or both; why?

- If you have ever tried to quit smoking in the past, what was the hardest part about quitting smoking for you? (e.g., physiological need, stress reliever, habit, etc.)

- What are the positive and negative aspects of smoking in your daily life?

- Do you think smoking affect males and females differently? How about particular age groups?

**Topic 2) Structure of a smoking cessation program**

- In your opinion, what would be the components of a practical and helpful smoking cessation program?

- Ideal format? (e.g., components you would add from previous programs)

- Ideal duration? (e.g., 6 months, a year, or longer)

- Group setting, individual based, or both?

- What makes a smoking cessation program effective? (e.g., family or professional support, educational features, etc.)

**Topic 3) The impact of smoking on health**

- Do you think there is any relationship between smoking and disease? If yes, can you provide some examples? If no, why not?

- If you have a relative or close friend who suffers from a health-related issue due to smoking, does this influence your smoking behavior? Can you elaborate?

- Have you received education about possible consequences of smoking? Which source(s) did you receive this information from? (e.g., GP, specialist, hospital, internet, etc.)

**Topic 4) The feasibility and application of alternative approaches to quitting, including e-cigarettes and telehealth**

4a) Vaping:

- Have you heard about e-cigarettes or vaping products?

- Do you think vaping is more or less harmful compared to tobacco cigarette smoking?

- Do you think vaping could be used as an alternative or replacement to tobacco cigarette smoking?

- Do you think using vaping as a smoking cessation method is a good idea?

4b) Telecommunication:

- Do you think a telecommunication approach (e.g., app, videos, skype, etc.) would be appropriate for smoking cessation? Why or why not?

- Do you think a combination of in-person interactions and telecommunication would be helpful for general health issues, including smoking cessation? Why or why not?

- Can you name some advantages and disadvantages with using telecommunication for general health issues?

- Have you used a telecommunication approach for your health issues? If yes, can you elaborate about your experience?
